# Piloting the Use of Patient-Specific Cardiac Models as a Novel Tool to Facilitate Communication During Cinical Consultations

**DOI:** 10.1007/s00246-017-1586-9

**Published:** 2017-02-18

**Authors:** Giovanni Biglino, Despina Koniordou, Marisa Gasparini, Claudio Capelli, Lindsay-Kay Leaver, Sachin Khambadkone, Silvia Schievano, Andrew M. Taylor, Jo Wray

**Affiliations:** 1 Bristol Heart Institute, School of Clinical Sciences, Bristol Royal Infirmary, University of Bristol, Upper Maudlin Street, Bristol, BS2 8HW UK; 2 Cardiorespiratory Division, Great Ormond Street Hospital for Children, NHS Foundation Trust, London, UK; 3 Institute of Cardiovascular Science, University College London, London, UK; 4 School of Medicine, King’s College London, London, UK

**Keywords:** Congenital heart disease, Rapid prototyping, Transition clinic, Communication, 3D printing

## Abstract

This pilot study aimed to assess the impact of using patient-specific three-dimensional (3D) models of congenital heart disease (CHD) during consultations with adolescent patients. Adolescent CHD patients (n = 20, age 15–18 years, 15 male) were asked to complete two questionnaires during a cardiology transition clinic at a specialist centre. The first questionnaire was completed just before routine consultation with the cardiologist, the second just after the consultation. During the consultation, each patient was presented with a 3D full heart model realised from their medical imaging data. The model was used by the cardiologist to point to main features of the CHD. Outcome measures included rating of health status, confidence in explaining their condition to others, name and features of their CHD (as a surrogate for CHD knowledge), impact of CHD on their lifestyle, satisfaction with previous/current visits, positive/negative features of the 3D model, and open-ended feedback. Significant improvements were registered in confidence in explaining their condition to others (p = 0.008), knowledge of CHD (p < 0.001) and patients’ satisfaction (p = 0.005). Descriptions of CHD and impact on lifestyle were more eloquent after seeing a 3D model. The majority of participants reported that models helped their understanding and improved their visit, with a non-negligible 30% of participants indicating that the model made them feel more anxious about their condition. Content analysis of open-ended feedback revealed an overall positive attitude of the participants toward 3D models. Clinical translation of 3D models of CHD for communication purposes warrants further exploration in larger studies.

## Introduction

Improvements in the treatment and follow-up of children with congenital heart disease (CHD) have resulted in a growing population of young people transitioning to adult care. Transition has received a lot of attention from professionals and researchers alike, not least because there is evidence that a significant proportion of patients never successfully transfer to adult services. Successful transition from paediatric to adult care requires engagement from the young person, their family and both paediatric and adult health care teams as the young person becomes increasingly autonomous [1–4]. Assuming responsibility for their health is part of that process, which necessitates a level of understanding and appreciation of what their condition entails and of what their cardiac anatomy looks like [5]. However, it is widely accepted that adolescents typically do not have adequate knowledge of their condition [6–9]. Education and knowledge about their CHD are key to achieving a successful transition to adult services, particularly as poor knowledge limits young people’s ability to communicate confidently with clinicians and thereby to engage fully with the health services they require [10–12]. In this context, patient–doctor communication is a complex process, essential to the delivery of high-quality care and directly impacting on patient satisfaction and adherence [13, 14]. Cardiology terminology, however, can be difficult to understand for the non-specialist and difficult for the specialist to explain in a non-patronising but understandable manner [15], and this can be further exacerbated by the invisibility of the cardiac lesion.

Technological advances in the realm of three-dimensional (3D) printing technology enable the manufacture of patient-specific models from medical imaging data. Recent work has shown the acceptability and feasibility of manufacturing CHD patient-specific models, reporting a positive experience for communicating with families in a clinical setting [16]. In this study, 3D models were well liked by parents and were considered to be more meaningful for the non-expert with respect to medical imaging data. Another recent study discussed the importance of involving all stakeholders in the technology (including patients and families) to evaluate the potential of 3D models for facilitating patient–doctor communication [17].

In this context, the present pilot study aimed to assess the impact of providing a patient-specific 3D model of their CHD to patients, during a transition clinic. We hypothesised that seeing and manipulating a patient-specific, 3D model would result in improved anatomical appreciation, improved ability of the young person to describe their CHD, and overall improved communication between the patient and the cardiologist.

## Materials and Methods

### Patients

The pilot study involved adolescent patients with CHD (n = 20, age range 15–18 years, 15 male) at the time of clinical consultation during a transition clinic. Patients presented with a range of CHDs: tetralogy of Fallot (n = 5), transposition of the great arteries (n = 5), aortic coarctation (n = 3), pulmonary atresia (n = 3), aortic stenosis with dilated ascending aorta (n = 2), double outlet right ventricle (n = 1), and Ebstein’s anomaly (n = 1). Young people were eligible to participate if they were aged between 14 and 18 years, had a primary diagnosis of CHD and had recent cardiac magnetic resonance (CMR) imaging data suitable for producing a patient-specific 3D model (see Intervention). Potential participants received an information sheet at home prior to their appointment, explaining what the study was about and that they might be approached to participate while in clinic. Written consent/assent, depending on participants’ age, was obtained prior to administering the survey and parents provided the consent for their child’s participation for those young people under 16 years of age. The study was approved by the National Ethics Research Service local committee (REC REF 13/LO/1569).

### Setting

The study involved completion of questionnaires, facilitated by a Research Administrator in the outpatient clinic of a specialist paediatric hospital, together with the presentation of unique 3D heart models to patients during their consultation with the clinician. The brief questionnaire could be completed on paper or an iPad and was administered before the clinical appointment, and repeated once the participating patients had seen their clinician and had been presented with their model during their consultation.

### Intervention

**Fig. 1:**
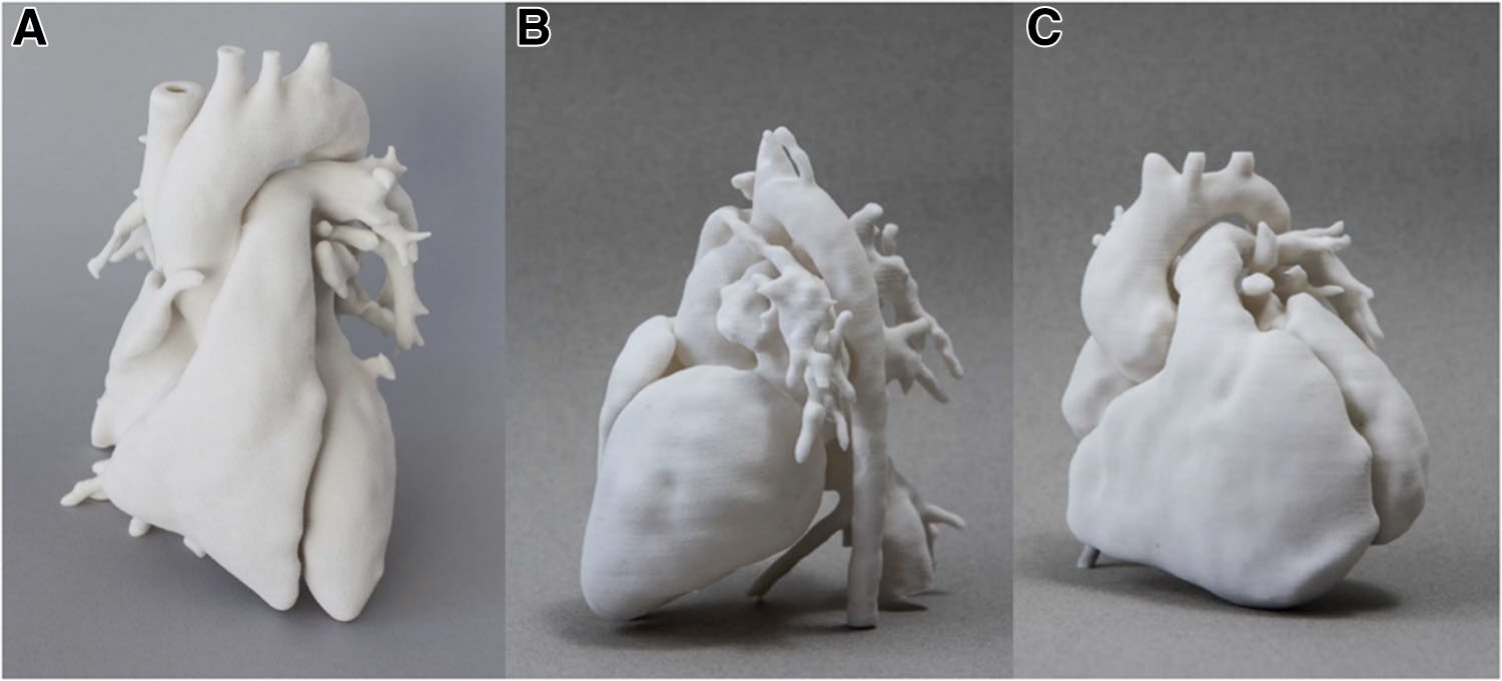
Example of 3D whole heart models manufactured by means of 3D printing, showing a normal heart from a healthy control for comparison purposes (**a**); a model of transposition of the great arteries repaired with arterial switch operation with Lecompte manoeuvre (**b**); a model of repaired tetralogy of Fallot (**c**)

A 3D model was created from CMR data of all identified potential study participants, according to the steps of image segmentation and volume rendering explained in detail elsewhere [18]. Models were generated from the 3D steady-state free precession (SSFP) whole heart sequence or from the angiogram sequence of the CMR examination, depending on image quality. All models were reconstructed by the same operator using commercial image-reconstruction software (Simpleware Ltd, Exeter, UK). Models included the whole heart and main vessels, except for cases of aortic coarctation and aortic stenosis with dilated ascending aorta, in which it was deemed more effective to highlight the area of interest (i.e. narrowing/ dilatation of the aorta) on a model including the left ventricle and the aorta to the level of the diaphragm (i.e. left side only). All models were printed with white nylon using selective laser sintering technology (3D Systems ZPrinters, Rock Hill, SC, USA). Examples of models from the study are shown in Fig. 1.

On the day of their clinic visit, patients were approached on arrival and, having provided consent, completed the first part of the questionnaire, focused on evaluating their perceived health status and their current knowledge of their CHD. Questions were 5-point Likert-type, yes/no or free text, and included ratings of health status, confidence in explaining their condition to others, name and features of the CHD, the effect of the CHD on their lifestyle and satisfaction with previous visits.

Patients subsequently saw their cardiologist for routine consultation and all were shown their 3D model, which was used to describe their anatomy and their CHD. A control model (i.e. full heart model with no CHD) was also provided to the cardiologist and it was up to his/her discretion to use it to further aid in the conversation.

At the end of their appointment and having held, observed and interacted about their 3D model, patients completed the second part of the questionnaire. All previous questions were asked again, in addition to an item enquiring as to their service satisfaction for the *current* visit. Patients were also asked to rate on a 5-point Likert scale their agreement with statements relating specifically to the model, including the 3D model “was fun”, “helped me understand my condition”, “improved this visit compared to previous ones”, and “made me more anxious about my condition”. They were also asked to name three good features and three things that could be improved about the model, and whether they would want to use it in future visits and would recommend it to a peer with CHD. Finally, the questionnaire offered participants an opportunity to provide additional feedback about the models and the service.

### Patient and Public Involvement

The survey was designed both for paper and for an iPad (iSurvey, harvestyourdata.com) based on feedback received from a parents’ focus group, indicating that the iPad could be more engaging for the adolescents. The survey was designed with a patient representative, to ensure readability, friendliness and appropriateness of the questions, suitability of the language, and overall length of the survey.

### Data Analysis

Each patient acted as his/ her own control, comparing differences before/after consultation, hence also indirectly comparing previous visits (i.e. no 3D model) with current visit (i.e. with 3D model). Results are presented as counts, proportions and mean ± standard deviation, where appropriate. Wilcoxon-signed rank tests were performed to compare responses of the two sections of the questionnaire, with p < 0.05 indicating statistical significance. Participants’ knowledge was quantified based on the correct name of the primary diagnosis, correct naming of associated keywords, and/or correct identification of features on diagrams, marking one point for each correct name, keyword or identified anatomical feature and summing the points. This was performed independently by two observers. Furthermore, content analysis was performed to analyse the free text responses provided by the participants.

## Results

The majority of participants (>75%) rated their health status as ‘well’ or ‘very well’ prior to their consultation.

**Fig. 2:**
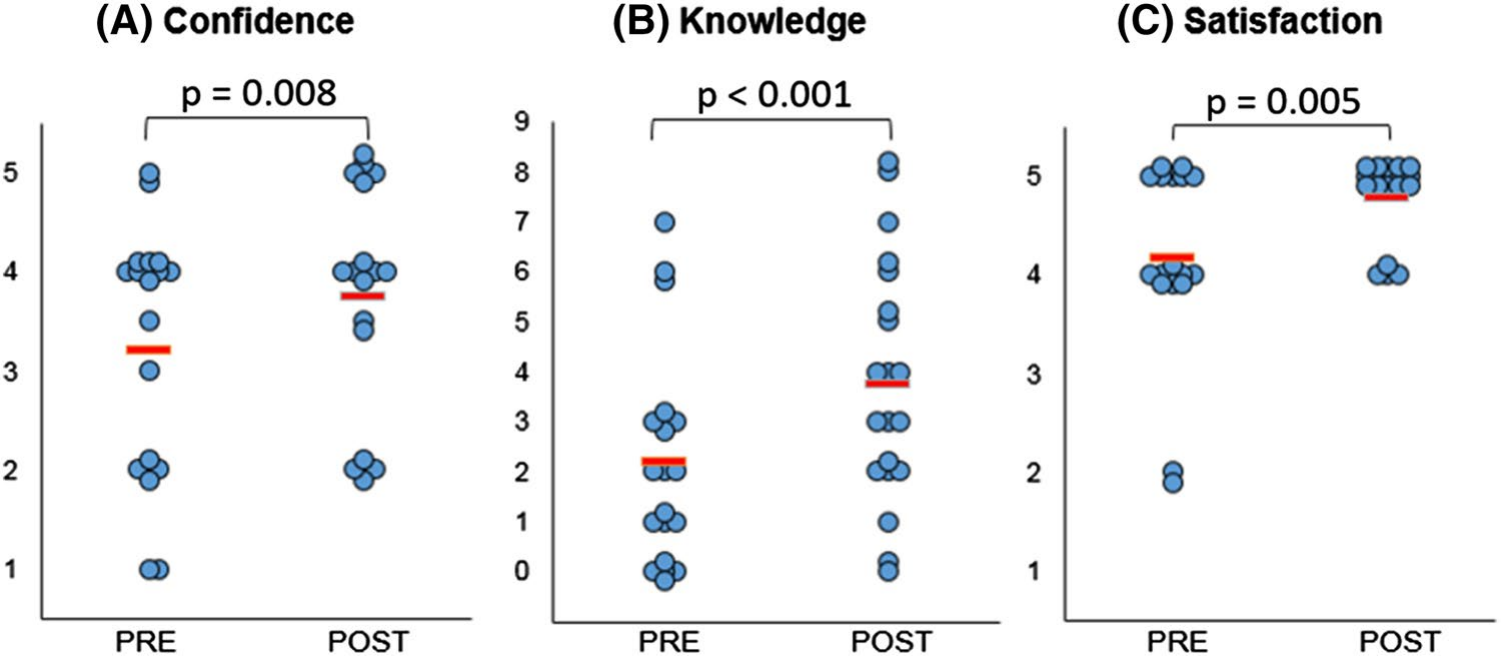
Statistically significant changes were observed in confidence (**a**), knowledge (**b**) and satisfaction (**c**) amongst participants comparing responses before (“Pre”) and after (“Post”) their consultation. Note for **a** 1 = Not at all confident – 5 = Very confident; for **b** each point represents a point in knowledge, as marked according to the correct name of primary diagnosis, correctly identified keywords and correct use of diagrams; for** c**1 = Very dissatisfied – 5 = Very satisfied. The *red lines* indicate average score

Confidence in explaining their condition to others significantly improved following the consultation with the 3D model (p = 0.008). Importantly, a significant (p < 0.001) improvement in knowledge was also registered after consultations. In no instance was a reduced knowledge score registered. The level of participant satisfaction following the visit improved significantly overall (p = 0.005), either increasing or remained constant. In no instance was satisfaction lower than for previous visits. These findings are summarised in Fig. 2.

Participants generally felt that their condition had little/no effect on their lifestyle and only half (n = 10) reported some limitations in taking part in sports activities. However, following their appointment, all participants (n = 20) mentioned lifestyle effects, providing much more eloquent replies, and reported limits in socialising, being asthmatic, not being able to get a tattoo or drinking alcohol socially, as well as limitations in playing rugby or running.

**Fig. 3:**
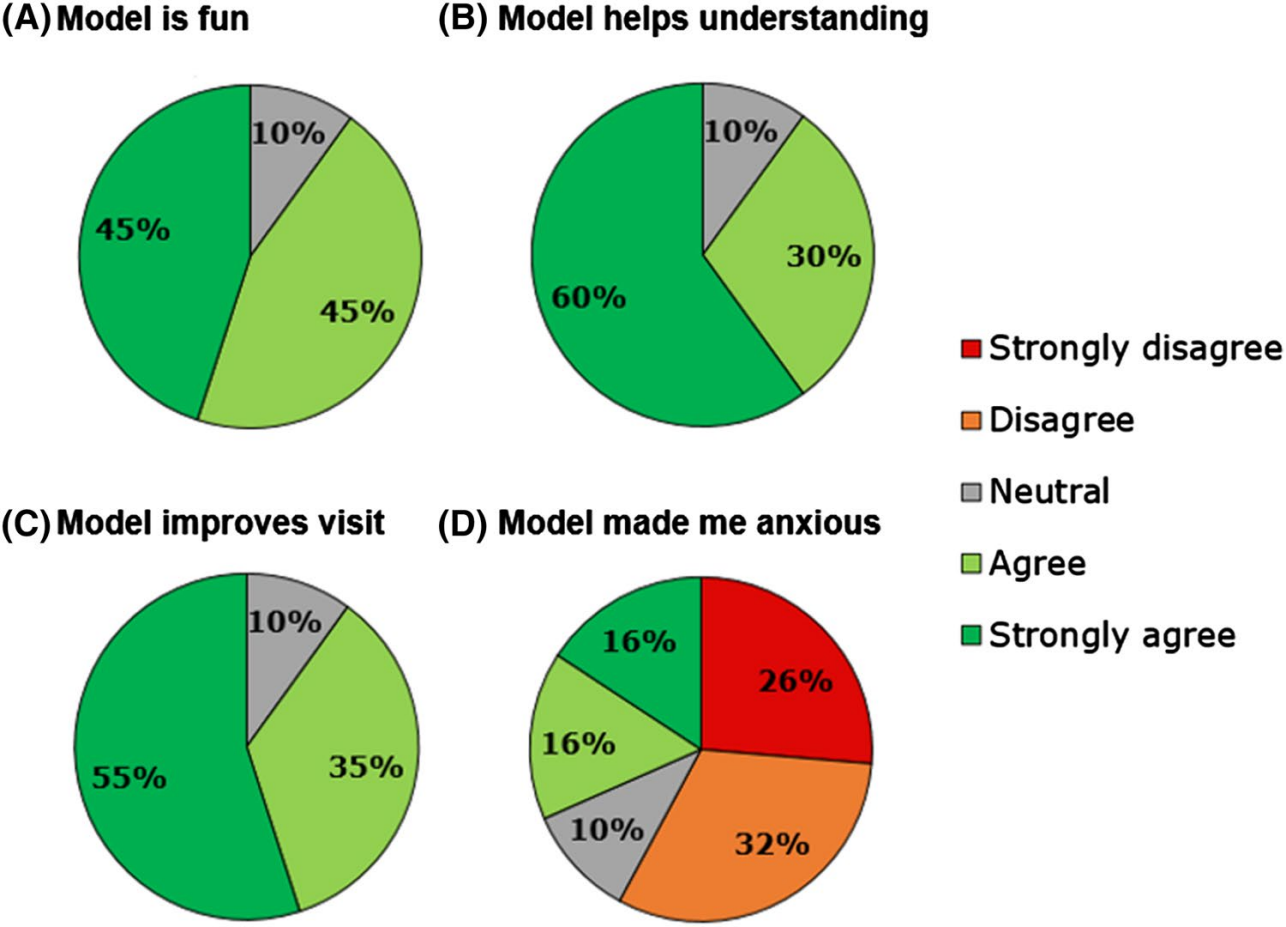
Summary of participants’ level of agreement to different statements on 3D models

The majority of participants reported that 3D models were fun and useful for their understanding, and a tool that improved their visit. A non-negligible 30% participants, however, indicated that the model made them feel more anxious about their condition (Fig. 3). Nevertheless, participants reported that they would want to have a 3D model for future visits and they would recommend it to a peer (Fig. 3).

Feedback from the young people was very informative (Table 1). Content analysis revealed that participants found models helpful in “*understanding [their] condition*” (n = 14) and that they were particularly impressed by viewing their *own* heart (n = 9) and by the *details* of the 3D models (n = 6). Models were generally commented on as “*interesting*” or “*helpful*” (n = 4) and particularly so for appreciating the *size* of the heart (n = 5).

**Table 1:** Young people’s feedback about the 3D models

“maybe a smaller [model] to take home to help explain to GPs the difference between a regular heart and my defect”
“…[the model] really helped to understand the significance of the surgery I’ve had. [It] helped visual[ise] the importance of being healthy.”
“….[the model] shows what my condition makes my heart look like”
“….[the model] helped [me] understand the way the blood flow[s]”
"Mum I would much rather get to take the model back, than a car, when I am 18"

## Discussion

Tailoring health messages in the context of a cardiac transition clinic can be facilitated by 3D printing technology, allowing the manufacturing of precise 3D replicas of each patient’s heart from medical imaging data (in this case CMR imaging, but also computed tomography). This pilot study sought to evaluate patient attitudes toward the models and the impact that seeing their own 3D heart model had at the time of consultation with their cardiologist. Evaluation of patient preference and model efficacy, in terms of potential knowledge improvement, are necessary steps toward demonstrating the clinical benefit of the technology and its potential for clinical translation.

Overall, patients responded very positively to the models, and questionnaire results indicated significant increases in both their confidence in explaining their condition to others as well as in their knowledge. Also, their narrative was considered to improve following their visit, based on the interpretation of improved descriptions of lifestyle limitations that had become more eloquent. The increase in articulate responses about how CHD may affect patient lifestyle in the follow-up questionnaire suggests an improvement in the participant self-narrative. Patients were better able to identify ways in which their lifestyles were affected, following exposure to 3D models. Interestingly, no reduction in knowledge was observed, which could indicate that seeing the 3D models did not generate confusion in the patients. On the other hand, it could be argued that the improved narrative is a result of the whole consultation, therefore suggesting that the models helped facilitate a conversation between the young person and the consultant, resulting in improved engagement, e.g. feeling more empowered to ask questions. Helping young people to take responsibility for their health is an important part of their transition to adulthood and increasing engagement, knowledge and confidence about their heart condition are key elements of that.

Whilst generally liking the models, some patients reported increased anxiety regarding their condition after their visit. This is not necessarily a negative feature, as it may indicate increased awareness as a result of the more in-depth conversation facilitated by the model. This may also suggest that a psychologist or adolescent nurse specialist should be involved in translating the technology clinically. Patient anxiety related to the use of the models should be addressed in future research, to determine how the models may be leading to increased anxiety and whether any increase is a direct or indirect consequence of using the models. Potentially, consultation with a psychologist or adolescent nurse specialist prior to participant selection could identify risk in some patients, and the possibility for 3D model use being counterproductive in some patients should be taken into consideration. Also, it is not known whether the anxiety level in these patients was actually linked to the information that they received during their consultation, as this was not explicitly asked in the questionnaire.

Participants provided valuable feedback indicating positive features of the 3D models with regards to understanding the anatomy and size of the heart, appreciating the personalised and unique quality of the model. In their feedback they also provided suggestions for improving the models if they were to use one again; in particular, half the respondents suggested the use of different colours which may be more familiar to them from other information sources such as pamphlets and books (e.g. red and blue vessels, typical textbook depiction of a heart). This could be helpful for distinguishing different parts of the heart and the vasculature more immediately. It was also suggested that different sizes should be explored (n = 2) and labels should be included on different parts of the models (n = 1).

From a methodological and logistical perspective, reconstruction of the imaging data would take a dedicated operator between one to two hours, and models could be printed within 48 h. The average cost of a model was £150, depending on the volume of the part. From a cost perspective, it could also be argued that if models were to facilitate the consultation process and result in improved patient adherence, the cost of a 3D model should be weighed against the cost of missing an appointment or non-adherence to other aspects of the treatment regimen. This remains speculative in the absence of an appropriate cost-effectiveness analysis. Nevertheless, recent evidence confirms that the proportion of young people lost to follow-up is a result of the need for formal transition programs, corroborated by the need for better education about CHD in adolescent patients [19].

Models can not only help making something invisible (i.e. the CHD) *visible*, but making something invisible *real*, allowing patients to view and manipulate an exact replica of their heart. As a visual aid, this can contribute substantially to personalising the health message, confirmed by the fact that some participants stressed in their feedback that they were impressed by viewing their *own* heart, rather than a generic or lesion-specific model. Models can also create a common ground between the young person and the cardiologist, removing some of the power imbalance from the consultation setting, in keeping with involving all stakeholders in the translation of the technology [17].

## Limitations

The study population is relatively small, limiting the generalisability of the results, and whilst it includes some complex cases of CHD it does not include any single ventricle patients, which would likely be another group of patients benefiting from seeing a model of their anatomy. The survey focused on young people’s response to the 3D models in a clinical setting, without any further follow-up, so the impact on future adherence to medical care or improved lifestyle cannot be predicted from these data. However, this will be the subject of future research.

## Conclusion

Adolescent cardiac patients appreciated 3D patient-specific models of their heart and vessels at the time of consultation during a transition clinic. A questionnaire indicated improvements in confidence, knowledge, narrative and patient experience, suggesting that clinical translation of 3D models for communication purposes warrants further exploration in larger studies.

## References

[1] Bhawra J, Toulany A, Cohen E, Moore Hepburn C, Guttmann A (2016). Primary care interventions to improve transition of youth with chronic health conditions from paediatric to adult healthcare: a systematic review. BMJ Open.

[2] Callahan TS, Winitzer RF, Keenan P (2001). Transition from pediatric to adult-oriented health care: a challenge for patients with chronic disease. Curr Opin Pediatr.

[3] Niwa K (2015). Adults with congenital heart disease transition. Curr Opin Pediatr.

[4] Somerville J (1989). Congenital heart disease in the adolescent. Arch Dis Child.

[5] McMurray R, Kendeall L, Parsons JM, Quirk J, Veldtman GR, Lewin RJP, Sloper P (2001). A life less ordinary: growing up and coping with congenital heart disease. Coronary Health Care.

[6] Wright M, Jarvis S, Wannamaker E, Cook D (1985). Congenital heart disease: functional abilities in young adults. Arch Phys Med Rehabil.

[7] Cetta F, Warnes CA (1995). Adults with congenital heart disease: patient knowledge of endocarditis prophylaxis. Mayo Clin Proc.

[8] Swann L, Hillis WS (2000). Exercise prescription in adults with congenital heart disease: a long way to go. Heart.

[9] Shebani SO, Miles HF, Simmons P, Stickley J, De Giovanni JV (2007). Awareness of risk of endocarditis associated with tattooing and body piercing among patients with congenital heart disease and paediatric cardiologists in the United Kingdom. Arch Dis Child.

[10] Canobbio MM (2001). Health care issues facing adolescents with congenital heart disease. J Pediatr Nursing.

[11] Reid GJ, Irvine MJ, McCrindle BW, Sananes R, Ritvo PG, Siu SC, Webb GD (2004). Prevalence and correlates of successful transfer from pediatric to adult care among a cohort of young adults with complex congenital heart defects. Pediatrics.

[12] Saidi A, Kovacs AH (2009). Developing a transition program from pediatric- to adult-focused cardiology care: practical considerations. Congenit Heart Dis.

[13] Ha JF, Longnecker N (2010). Doctor-patient communication: a review. Ochsner J.

[14] Martin LR, Williams SL, Haskard KB, Dimatteo MR (2005). The challenge of patient adherence. Ther Clin Risk Manag.

[15] Blackman J, Sahebjalal M (2014). Patient understanding of frequently used cardiology terminology. Br. J Cardiol.

[16] Biglino G, Capelli C, Wray J (2015). 3D-manufactured patient-specific models of congenital heart defects for communication in clinical practice: feasibility and acceptability. BMJ Open.

[17] Biglino G, Capelli C, Leaver LK, Schievano S, Taylor AM, Wray J (2015) Involving the public and medical staff in the evaluation of 3D printing models of congenital heart disease. Commun Med 12:157–169 10.1558/cam.28455 29048144

[18] Schievano S, Migliavacca F, Coats L (2007). Percutaneous pulmonary valve implantation based on rapid prototyping of right ventricular outflow tract and pulmonary trunk from MR data. Radiology.

[19] Heery E, Sheehan AM, While AE, Coyne I (2015). Experiences and outcomes of transition from pediatric to adult health care services for young people with congenital heart disease: a systematic review. Congenit Heart Dis.

